# Characterization of food waste-driven carbon dot focusing on chemical structural, electron relaxation behavior and Fe^3+^ selective sensing

**DOI:** 10.1016/j.dib.2019.104038

**Published:** 2019-05-23

**Authors:** Jungbin Ahn, Younghan Song, Ji Eon Kwon, Jeongyeon Woo, Hyungsup Kim

**Affiliations:** a Department of Organic and Nano System Engineering, Konkuk University, Seoul, 05029, Republic of Korea; b Department of Materials Science and Engineering, Seoul National University, Seoul, 08826, Republic of Korea

**Keywords:** Carbon dots, Electron relaxation behavior, Chemical structural analysis, Fe^3+^ quenching

## Abstract

In the study, carbon dot (CD) with high fluorescence properties was obtained via one-step hydrothermal carbonization of food model and sandwich leftover, respectively. The data in the article represent the change of the chemical structure and PL properties of the food waste-driven carbon dot (FWCDs). In higher carbonization temperature, pyridinic N and graphitic N were increased while amino N and pyrrolic N was decreased. The lifetime was increased with the increase of temperature. The CD prepared from sandwich leftover showed the dependency of the emission on the exciting wavelength and excellent Fe^3+^ sensitivity without significant change of lifetime. It also had a pH-sensitive fluorescence feature and good stability in NaCl solutions. For more insight, please see Food waste-driven N-doped carbon dots: Applications for Fe^3+^ sensing and cell imaging Ahn et al., 2019.

**Table:** Specifications table

Subject area	*Physics, Chemistry, Material science*
More specific subject area	*Photoluminescence, Carbon-based Nanomaterial,*
Type of data	*Table, figure, graph, image*
How data was acquired	*XPS (K-alpha, Thermo Scientific), TCSPC (Fluo Time 200 instrument, Picoquant), Digital camera (G10, Canon), PL spectrometer (FS-2, SICNCO), UV spectrometer (Cary 60 UV/vis spectrophotometer, Agilent Technologies)*
Data format	*Raw, analyzed*
Experimental factors	*Carbon dots were synthesized using food wastes via one-step hydrothermal carbonization*
Experimental features	*Food waste-driven carbon dots were characterized with XPS, TCSPC, PL and UV spectrometer*
Data source location	*Seoul, Republic of Korea*
Data accessibility	*Physics, Chemistry, Material science*
Related research article	*J. Ahn, Y. Song, J. E. Kwon, S. H. Lee, K. S. Park, S. Kim, J. Woo and H. Kim, Food waste-driven N-doped carbon dots: Applications for Fe*^*3+*^*sensing and cell imaging, Materials Science & Engineering C 102 (**2019**) 106–112.*

**Table:** 

**Value of the data** • This data can help for the understanding of structural change of CD during hydrothermal carbonization of complex mixture including food waste. • The changes of the chemical structure and the electron relaxation behavior along the carbonization temperature is beneficial to study the photoluminescence mechanism of carbon dots. • The data exhibit the possibility of the prepared carbon dot for Fe^3+^ sensing with high selectivity in the presence of other metal ion.

## 1 Data

Nanomaterials with fluorescence properties including carbon dots are having great attention due to its wide application area such as metal ion sensing and biological imaging [1], [2], [3]. Herein, we synthesized carbon dots with food waste-driven cat feed stock and sandwich leftover.

**Fig. 1:**
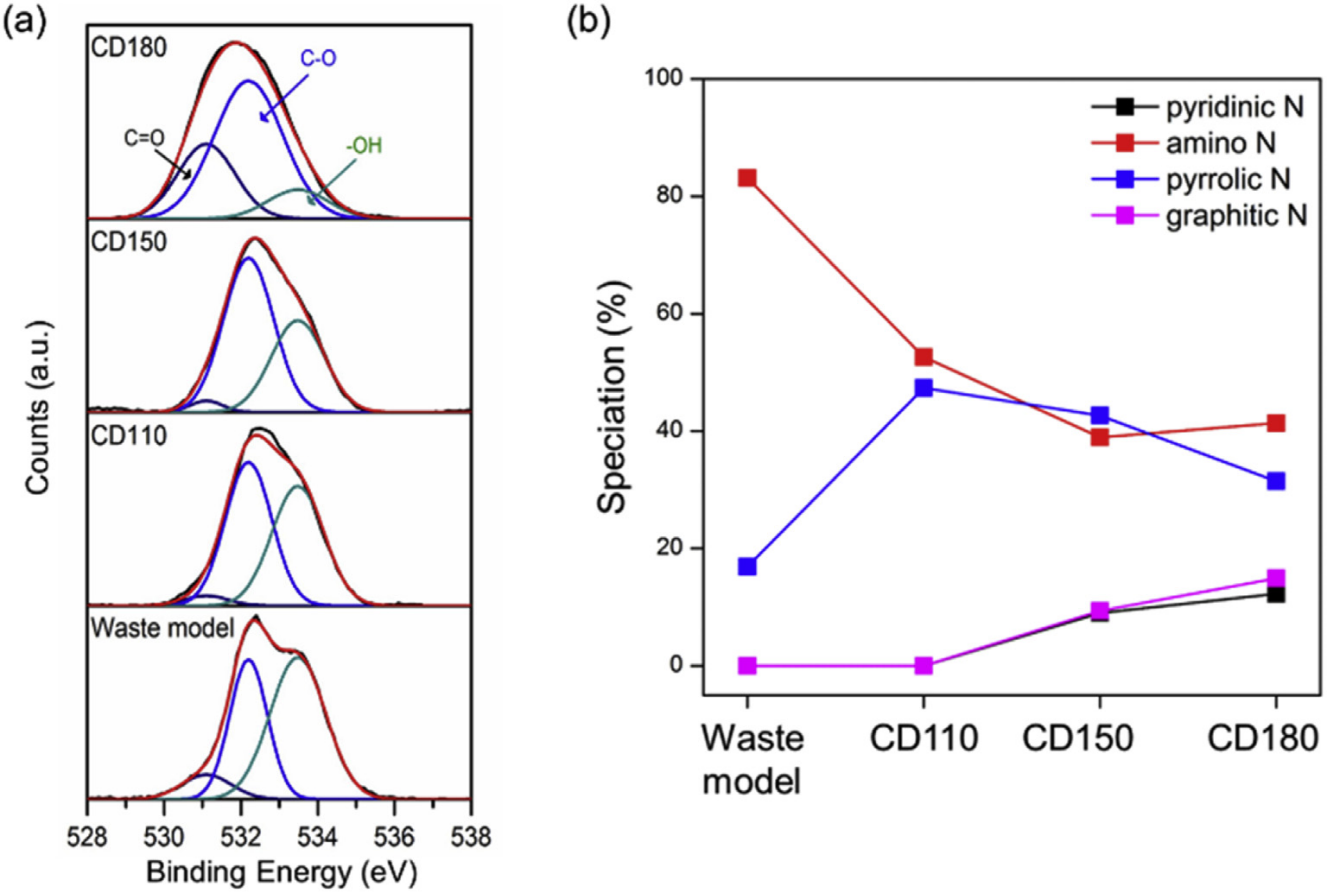
(a) High resolution XPS spectra of O1s and (b) the speciation of the nitrogen bonding of the samples.

Fig. 1 shows the chemical structure changes of FWCDs along the carbonization temperature. As the temperature increased, the peak for –OH bonding was decreased while the peaks for C–O and C=O bonding were increased (Fig. 1a). Fig. 1b shows the ratio of each nitrogen speciation in the synthesized CDs, which indicate the structure of N-containing aromatic compounds were more developed along the temperature. The TEM images of synthesized CDs their size distribution can be seen in Fig. 1 of [1].

**Fig. 2:**
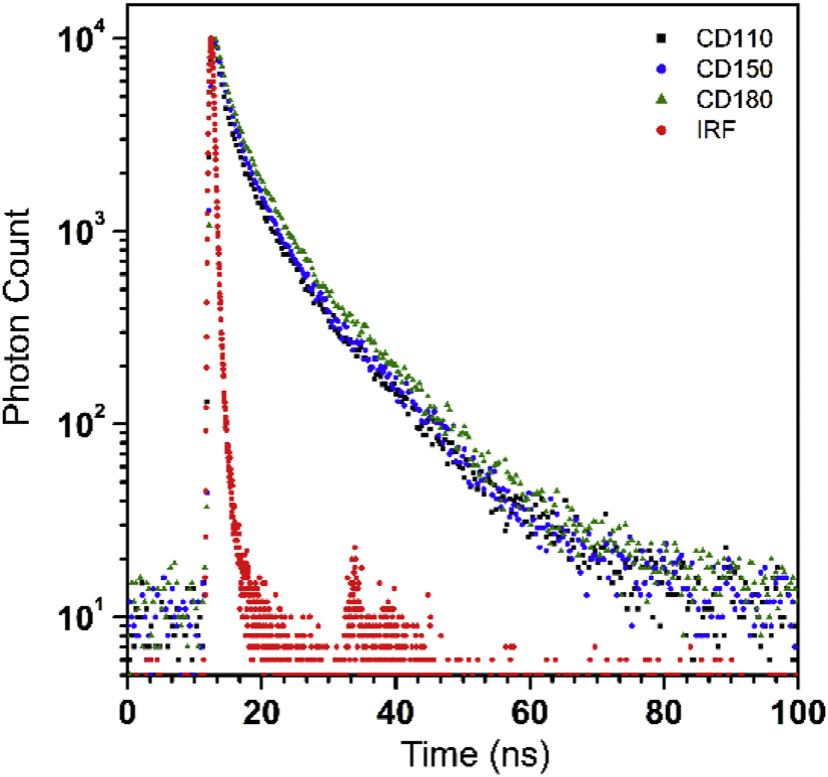
Decay curves of the CDs collected at emission wavelength of the individual maximum intensity.

The Fig. 2 shows the lifetime decay curves of the CDs and were interpreted in terms of a tri-exponential function:

(1) $$I\;\left(t\right)=\int_{-\infty }^{t}IRF\left(t^{'}\right)\sum_{i\;=1}^{n}A_{i}e^{-\frac{t-t^{'}}{\tau_{i}}}dt^{'}$$

Where A_i_ and τ_i_ are the amplitude and the decay times of lifetimes, respectively. All the curves were well-fitted with χ^2^ value below 1.1.

Radiative lifetime was can be calculated from the average lifetime (τ_av_) and the fluorescence quantum yield (φ) using Equation (2). The lifetime and the radiative/non-radiative recombination rate are finally obtained from the radiative and non-radiative lifetimes using Equations (3), (4) (Table 1 of [1])

(2) $$\varphi =\frac{\tau_{av}}{\tau_{r}}$$

(3) $$\frac{1}{\;\;\tau_{av}}=\frac{1}{\tau_{r}}+\frac{1}{\tau_{nr}}$$

(4) $$k_{r}=\frac{1}{\tau_{r}},\;k_{nr}=\frac{1}{\tau_{nr}}$$

where φ = fluorescence quantum yield, $\tau_{av}$ = average lifetime, $\tau_{r}$ = radiative lifetime, $\tau_{nr}$ = non-radiative lifetime, $k_{r}$ = radiative recombination rate constant and $k_{nr}$ = non-radiative recombination rate constant.

**Table 1:** Functional groups identified from FT-IR spectra of the samples.

Wavenumber (cm^−1^)	Types of vibration	Functional groups	References in the article
3400–3200	Stretching	-OH, –NH	[5]
2926	Asymmetrical stretching	C–H	[5]
2857	Symmetrical stretching	C–H	[5]
1657	Stretching	C=O (Amide I)	[6]
1640	Stretching	C=O	[6]
1580	Bending, stretching	-NH, –NH (Amide II)	[5], [6]
1400	Stretching	C–N	[7]
1050	Stretching	C–O	[8]
872, 800	Out-of-plane bending	C–H of phenazine skeleton	[9]

The functional groups of waste model and CDs were summarized in Table 1.

**Fig. 3:**
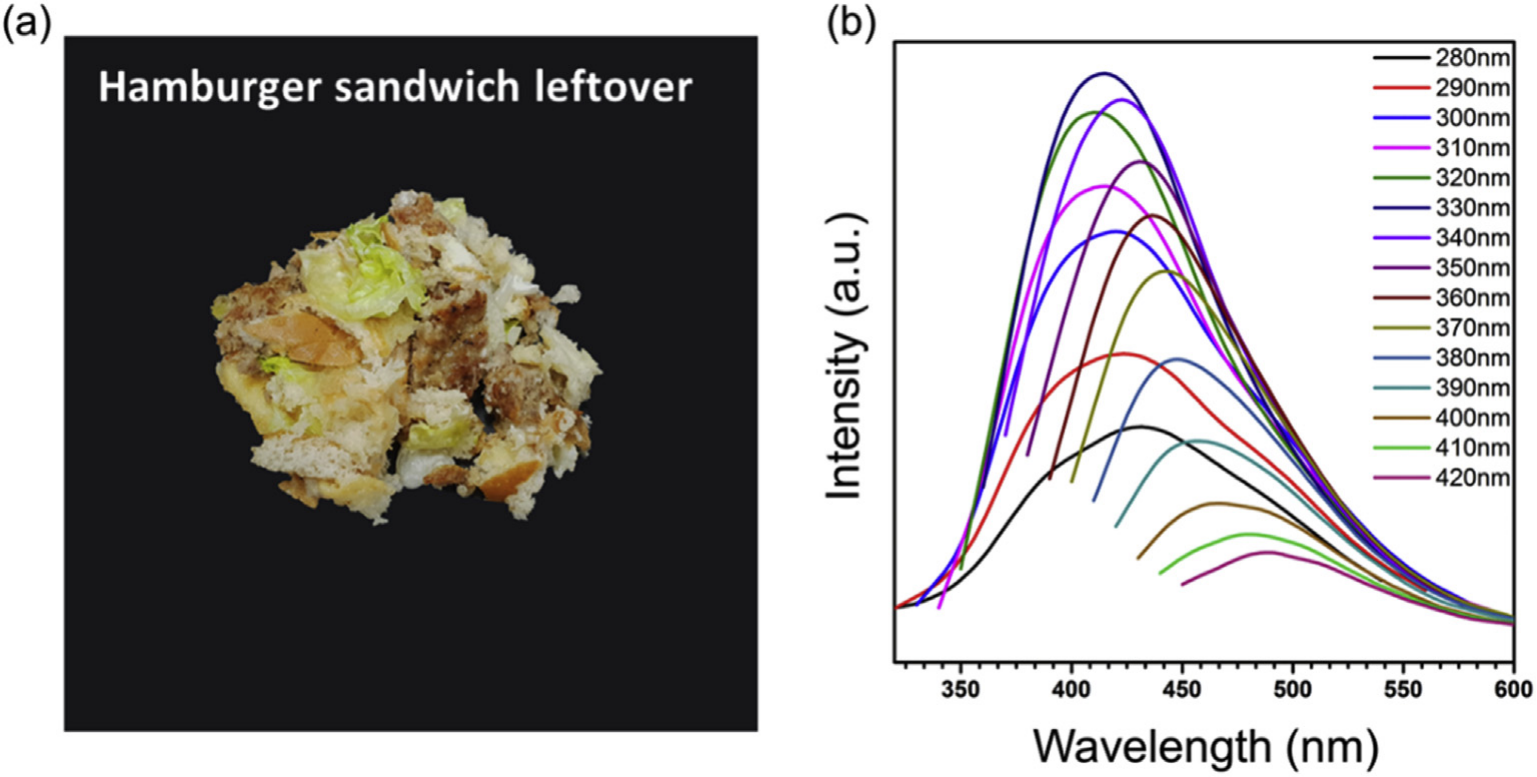
(a) The digital image of precursors for FWCDs and (b) emission peaks excited by each wavelength, which indicated by individual colors.

Fig. 3 shows the image of used sandwich leftover and PL spectra of synthesized FWCDs. The TEM images of FWCDs can be seen in Fig. 5a of [1].

**Fig. 5:**
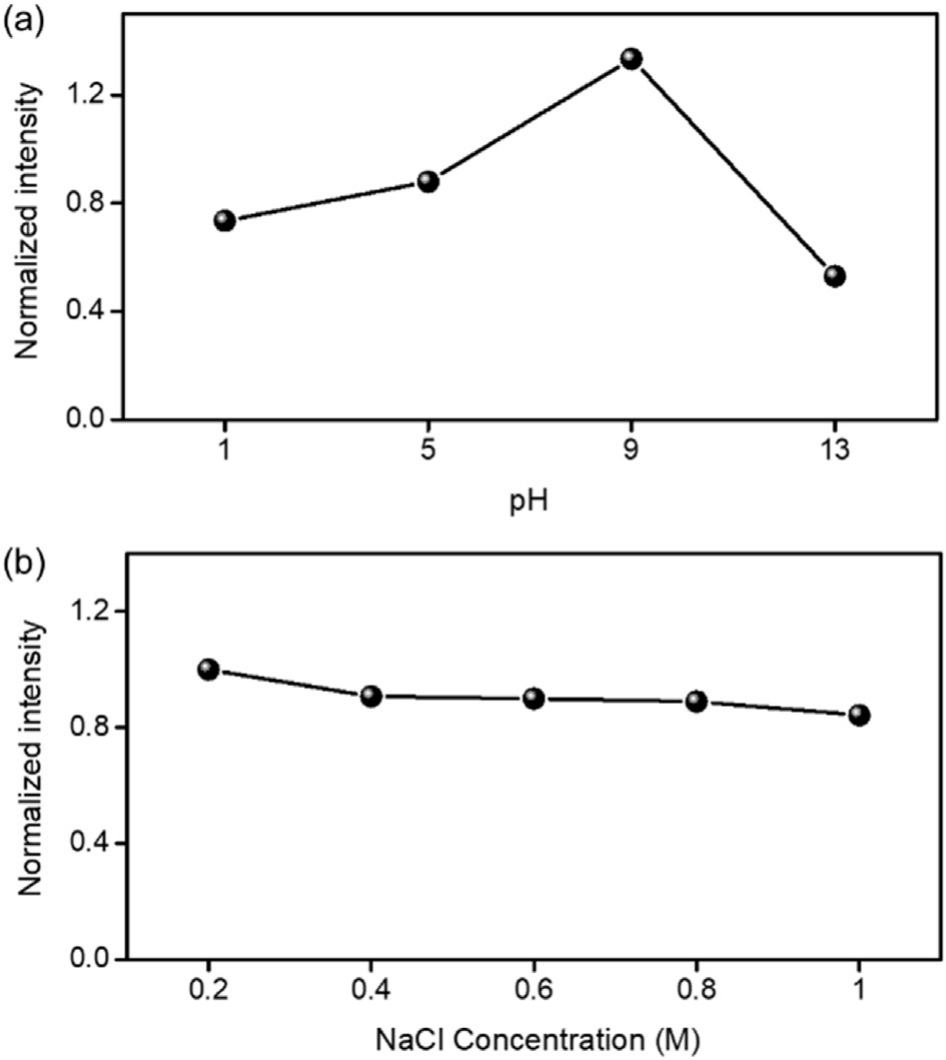
The effects of different (a) pH and (b) NaCl concentrations to the fluorescence intensity of FWCDs solution.

**Fig. 4:**
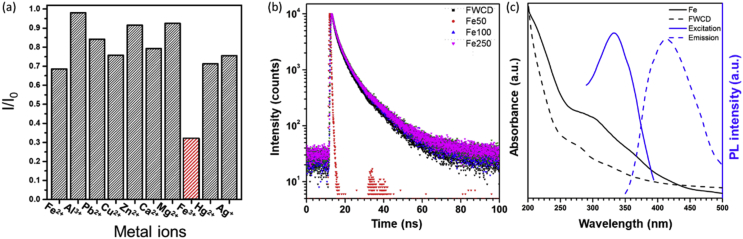
(a) Plot of relative fluorescence intensity of FWCDs solution in different metal solutions. (b) Fluorescence decay curves of FWCDs in the absence and presence of Fe^3+^ under excitation of 342 nm. Fe50, Fe100 and Fe250 refer lifetime decay of FWCDs in the different Fe^3+^ concentrations of 50, 100 and 250 μM, respectively. (c) UV–vis absorption spectra of Fe^3+^ and FWCDs, and photoluminescence excitation/emission curves of FWCD.

The FWCDs showed the selective sensing capability for Fe^3+^. In Fig. 4a, the fluorescence intensity of FWCDs solutions was significantly decreased in the presence of Fe^3+^ while other metal ions insignificantly influenced on the PL. The quenching mechanism of FWCDs was characterized by Time-correlated single photon counting (TCSPC), UV-vis spectrometer and PL spectrometer. Fig. 4b shows the fluorescence decays of the FWCDs quenched by Fe^3+^. The obtained values were summarized in Table 2. The average lifetime of FWCDs was slightly increased in Fe^3+^solutions, However, the lifetime decay of FWCDs in Fe^3+^ solution was not changed along Fe^3+^ concentration. The identical lifetime indicates that the energy transfer between Fe^3+^ and FWCDs did not occur in the quenching process, known as Inner Filter Effect (IFE) [4]. In Fig. 4c, the typical feature of IFE behavior was shown by the overlapping curves of the absorption band of Fe^3+^ in UV spectra and emission or excitation bands of FWCDs in PL spectra. Table 3 are the list of comparing the detection of Fe^3+^ with carbon dots prepared from various biomass-based sources. Fig. 5 exhibits the FWCDs had a pH-sensitive fluorescence feature and good stability in NaCl solutions.

**Table 2:** Photoluminescence lifetime ($\tau_{1},\;\tau_{2},\;\tau_{3}\;and\;\tau_{av})$ of the FWCDs, with and without Fe^3+^.

	FWCDs	Fe50	Fe100	Fe250
$\tau_{1}\;$(ns)	11.55	11.99	11.93	11.75
$\tau_{2}\;$(ns)	4.19	4.34	4.20	4.26
$\tau_{3}\;$(ns)	1.16	1.20	1.16	1.18
$\tau_{av}\;$(ns)	2.79	3.14	3.24	3.17

**Table 3:** Comparison of limit of detection (LOD) and linear detection range for Fe^3+^ of carbon dots prepared from various biomass-based sources.

References	Carbon source	LOD (μM)	Linear detection range (μM)
[10]	Used black tea	0.25	0.25–60
[11]	Sweet potato	0.32	1–100
[12]	Bergamot	0.075	0.025–100
[13]	Silkworm	0.2	1–500
[14]	Sugarcane molasses	1.46	1–100
[15]	Onion waste	0.31	0–20
[16]	Curcumin	0.62	0–6
This work	Hamburger sandwich leftover	32	12.5–100

## 2 Experimental design, materials and methods

### 2.1 Synthesis of carbon dots

Cat feed stocks (Catsrang, Dajoo industry) produced from the organic waste were used for the synthesis of CDs along the temperature. The feed stocks were ground to fine powder and dried at 65 °C for 24 h. After the powder was mixed with 50ml distilled water for 7 wt%, hydrothermal carbonization of the source was conducted at 110, 150 and 180 °C for 24 h. The obtained solution was purified by filtering (0.5 μm PTFE membrane) and dialysis (Biotech CD dialysis tubing, 0.5–1.0 kDa, Spectrum Labs.). The carbon dots obtained at 110, 150 and 180 °C were noted as CD110, CD150 and CD180, respectively. Hamburger sandwich leftover was synthesized at 180 °C to food waste-driven carbon dots.

### 2.2 Selectivity test for Fe^3+^

The all metal salts in this experiment were purchased from Sigma Aldrich. And they was used as received.

For metal quenching test, the all metal salts were dissolved at concentration of 500 μM and mixed with FWCDs solution (10 μg/ml). The PL intensities were measured after 30 min by excitation wavelength of 340 nm.

### 2.3 Characterization

X-ray photoelectron spectroscopy (XPS, K-alpha, Thermo Scientific) were carried out to characterize the chemical structure of the samples. Fluorescence lifetimes were obtained by the time-correlated single photon counting method (TCSPC, Fluo Time 200 instrument, Picoquant). An excitation source was used 342 nm pulsed LED with repetition rate of 5 MHz. The decay profiles were analyzed by FluoFit Pro software using exponential fitting models through deconvolution with instrumental response functions (IRF).

The PL spectroscopy (FS-2, SICNCO) and UV–vis absorption spectroscopy (Cary 60 UV/vis spectrophotometer, Agilent Technologies) were used for PL properties.
