# Supplementary material for: Characterization of food waste-driven carbon dot focusing on chemical structural, electron relaxation behavior and Fe3+ selective sensing
Source: Data Brief. 2019 May 23;25:104038. doi: 10.1016/j.dib.2019.104038 (PMC6554359; doi:10.1016/j.dib.2019.104038)
Supplement: Multimedia component 1 [file mmc1.docx]

Dear Editor in Chief, Hao-Ran Wang and Ganhui Lan of ***Data in Brief***

The authors of the manuscript “Characterization of Food Waste-Driven Carbon Dot Focusing on Chemical Structural, Electron Relaxation Behavior and Fe^3+^ Selective Sensing” by Jungbin Ahn, Younghan Song, Ji Eon Kwon, Jeongyeon Woo and Hyungsup Kim declare that there is no conflict of interest.
